# Reconstructing the invention of the wheel using computational structural analysis and design

**DOI:** 10.1098/rsos.240373

**Published:** 2024-10-23

**Authors:** Lee R. Alacoque, Richard W. Bulliet, Kai A. James

**Affiliations:** ^1^ Department of Aerospace Engineering, University of Illinois Urbana-Champaign, Urbana, IL, USA; ^2^ Department of History, Columbia University, New York NY, USA; ^3^ School of Aerospace Engineering, Georgia Institute of Technology, Atlanta, GA, USA

**Keywords:** computational solid mechanics, computational design, history of technology

## Abstract

The invention of the wheel is widely credited as a pivotal moment in human history, yet the details surrounding its discovery are shrouded in mystery. There remains no scholarly consensus on key questions such as where, how and by whom this technology was originally invented. In this study, we employ state-of-the-art techniques from computational structural mechanics to shed light on this long-standing puzzle. Based on this analysis, we propose a probable path along which the wheel evolved via a sequence of three major innovations. We also introduce an original computational design algorithm that autonomously generates a wheel-and-axle system using an evolutionary process that offers insight into the way in which the first wheels likely evolved nearly 6000 years ago. Our analysis provides new supporting evidence for the recently advanced theory that the wheel was invented by Neolithic miners harvesting copper ore from the Carpathian Mountains as early as 3900 BC. Moreover, we show how the discovery of the wheel was made possible by the unique physical features of the mine environment, whose impact was analogous to the selective environmental pressures that drive biological evolution.

## 1. Introduction

Over the course of human history, the details surrounding many seminal events have been lost to time. The invention of the wheel is an example of one such episode about which we know very little. The wheel has been described as the most important mechanical invention of all time [1] and has been credited with creating seismic social and economic shifts in the trajectory of human history [2]. Yet, surprisingly little is known about the origin of this revolutionary technology. Conventional tools, such as carbon dating, have provided an approximate time frame for the discovery, and identified several candidate civilizations as potential inventors [3], but thus far there remains no scholarly consensus on key questions like where, how and by whom the wheel was originally discovered.

In this study, we use techniques from computational analysis and design to provide insights into these questions. Design science seeks to uncover connections between the structure and function of engineered systems. Typically, the designer’s role is to create a system that is best suited to facilitate a prescribed task. In the case of mechanical systems, we use physics-based computational models to analyse and design structures and mechanisms [4]. Here, we turn this process on its head. We begin with a known description of the structure of an engineered system—in this case, ancient wheel systems taken from the archaeological record—and using the techniques of computational solid mechanics and computational design, we deduce new knowledge about the precise function of the system, including specific advantages and disadvantages conferred by its unique physical attributes. In this way, our methods serve as a forensic tool that can be used alongside established approaches like carbon dating and palaeolinguistics [2,5] to learn about the circumstances from which this ancient technology arose.

Our physics-based analysis uses computational mechanics to model the elastic response and stress distribution of the wheel structure. This analysis framework is then used to power an original design algorithm, which is based on the topology optimization method [6]. The algorithm contains a mathematical description of the mechanical function that the design must ultimately perform, along with a mathematical model describing the physics that governs the structure and its constituent materials. Based on this information, the algorithm automatically synthesizes a wheel-and-axle structure despite being given little prior information about the system’s geometry. In this way, we simulate a plausible path along which the evolution of a wheel-and-axle can proceed naturally. Along this evolutionary pathway, each new design yields incrementally improved performance over its predecessor. To achieve this capability, our algorithm contains several novel features and mathematical formulations. These include a design-dependent contact loading formulation to model the forces acting on the axle. The algorithm is able to generate designs of axisymmetric structures with an orthotropic material model. This choice of material model reflects the fact that the original inventors of the wheel probably used wood as a primary design material [3].

## 2. A new theory of how the wheel evolved

**Figure 1.**
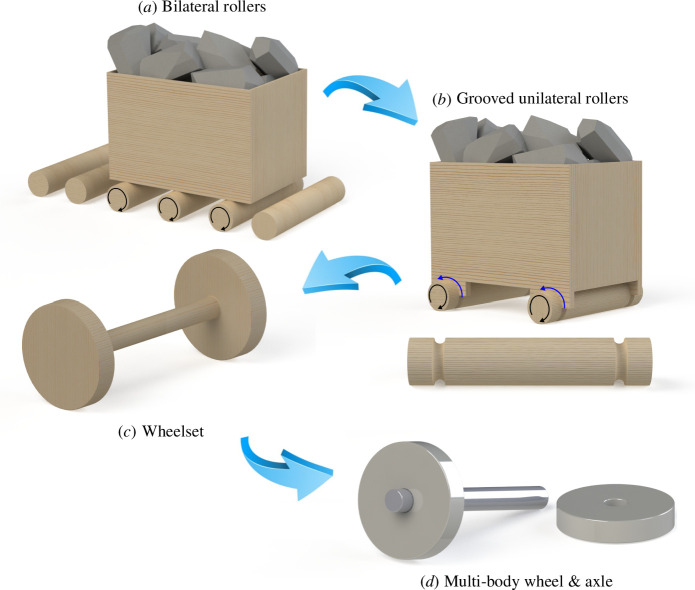
Evolution of the wheel-and-axle system. (*a*) Frictionless bilateral rolling with two spent rollers shown on the left of the image, (*b*) unilateral rolling with grooved rollers held in place by semi-circular sockets, and blue arrows indicating sliding at the friction surface, (*c*) a wheelset with two spokeless wheels fixed to the ends of a slender axle forming a monolithic structure and (*d*) a partially assembled multi-body wheel-and-axle system in which the wheels rotate independently of the axle.

Some scholars and antiquarians have long suspected that the wheel evolved from free rollers [1,7–9], while others have expressed scepticism about this theory [10,11]. Here, we propose a step-by-step path and physics-based rationale for how and why this evolution took place. We use the term *free rollers* to refer to a series of untethered cylinders, poles or tree trunks that are placed on the ground evenly spaced and perpendicular to the direction of transport. The cargo being transported would rest upon this array of rollers and be pushed or pulled forward as the rollers rolled along the ground. This process is illustrated in figure 1*a*. When used successfully, rollers could eliminate sliding, thereby reducing friction losses.

Despite the reduced friction, rollers have a major disadvantage in that they become useless once the cargo passes over them. One must either place rollers along the full length of the path to be travelled (which is unrealistic for most distances), or one must replenish the spent rollers by bringing them around to the front of the rolling path. Both approaches would have been impractical within a mine, which is generally accessed via a narrow, human-made trench or tunnel. Instead, it appears the miners found a way to adapt the roller concept to suit their specific circumstances. Adding sockets to the bottom of the vessel containing the cargo allowed the rollers to sit inside the sockets, forming a rudimentary cart. In this way, as the container was pulled forward, the rollers would be pulled along with it (see figure 1*b*). Hereafter, we refer to this process as *unilateral* (one-sided) rolling, since the rollers undergo rolling on only one side of their circumference, while sliding occurs at the roller-socket interface. By contrast, standard bilateral rollers have two rolling surfaces, and experience little or no sliding. The use of unilateral rolling represented a trade-off. It introduced some friction due to the presence of the sliding surface but there was no need to replenish spent rollers. This was an essential advantage since it allowed the cargo to span the full width of the mine tunnel, thereby demanding fewer trips to and from the source of the ore. Also, passageways could remain narrow, thus requiring less labour to build. Unilateral rolling also meant that the rollers were less likely to slip out of alignment, which necessitated periodic manual readjustment, as has been reported with the use of bilateral rollers [12]. So while the adoption of unilateral rollers imposed some cost in the form of increased friction, this strategy conferred significant advantages over free rollers, since it eliminated the need for periodic realignment and replenishing of rollers. Note also that when operating inside a tunnel, spent rollers could not simply be rolled around to the front of the cart but rather they would have to be lifted and carried or dragged, which would require additional energy expenditure.

**Figure 2.**
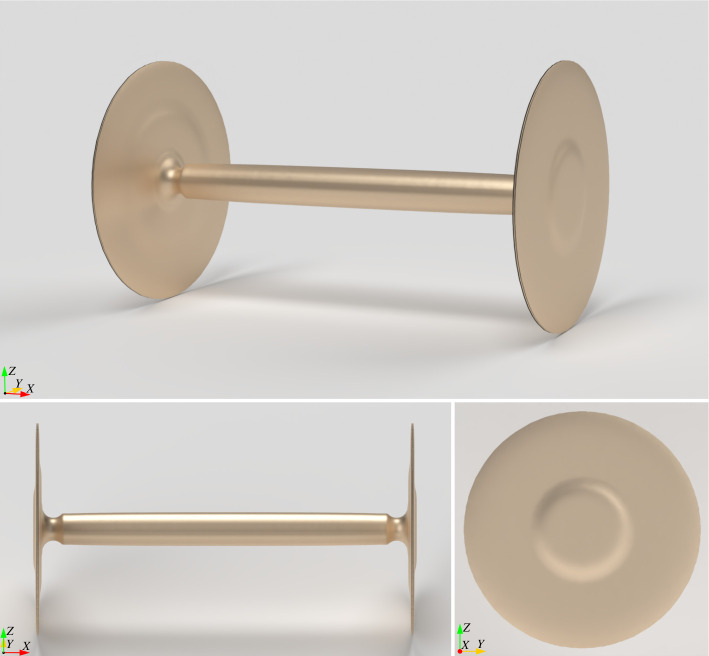
Geometry of the computationally generated wheelset structure.

Since unilateral rolling reintroduces a friction surface, it may appear as though this would be the mechanical equivalent of simply dragging the cart on skids, which was a commonly used method of transport throughout the ancient world [10,13]. However, bilateral rolling offers two significant advantages over dragging. First, by transferring the friction surface from the roller–ground interface to the roller–cart interface, one would have control over both frictional surfaces. While cart operators would have been limited in their ability to process the surface of the ground to reduce roughness and friction, they could easily process the surfaces of both the roller and the cart in order to reduce the coefficient of friction. The second advantage of limiting friction to the wheel–cart interface is that the cart operators could now apply lubricant to the friction surface, thereby significantly reducing the friction force. For this reason, the presence of grooves on the roller surface is highly significant. Grooves would have provided a channel in which to apply lubricant where it could remain free from contact with the ground. Whereas grooveless rollers would summarily deposit most of their lubricant on the ground after a few revolutions, a grooved roller could retain its lubricant for a much longer duration.

Wheel historians have long theorized about the use of grooved rollers [1,14]. In his 2011 treatise on the history of rotating machinery, Rao surmises that grooves initially formed inadvertently from the indentation of the rollers caused by narrow sledges that sat between the rollers and the heavy cargo being transported [1]. In his 2016 book on the history of the wheel, Bulliet observes that this theory is ubiquitous, but as yet unsubstantiated by archaeological evidence [14]. Whether their introduction was incidental or deliberate, these grooves set in motion the next stage of the evolution, which ultimately led to the familiar wheel-and-axle configuration. We propose that following the introduction of grooved unilateral rolling, there was an expansion and coalescing of the roller’s grooves to form a single channel in the centre of the roller (see figure 1*c*). It is possible that this shape change was initially motivated by the desire for clearance so that the cart could easily pass over small objects obstructing its path.

By creating this large channel in the centre of the roller, designers effectively forged a central axle. In addition to providing clearance, this modification yielded a lighter more portable roller, while preserving structural stiffness. But the most significant benefit of the narrow axle was mechanical advantage. Because of this principle, an axle whose diameter was one-tenth that of the wheel would require roughly one-tenth the pushing force needed for a prismatic roller with no axle. Our theory differs from earlier theories in that previous authors did not address the transition to unilateral rolling, nor did they relate the evolution of the axle to mechanical advantage.

In §4, we derive an equation that quantifies mechanical advantage and describes the required pushing force as a function of the axle radius. Next, we simulate the evolution of the wheel-and-axle structure from a unilateral roller using a structural optimization algorithm in which the objective function (also known as the *cost* function) is the mechanical advantage formula we previously derived. In keeping with the forensic nature of our study, the investigation begins with the wheel-and-axle geometry as the starting point. We then attempt to identify a set of boundary conditions, a design objective and an initial baseline design (i.e. the initial roller design from which the wheel evolved) that would cause the algorithm to independently arrive at the wheel-and-axle geometry. Therefore, the algorithm, which comprises just one part within our broader investigation, has no explicit prior knowledge of the wheel-and-axle concept. Instead, it is provided only with a mathematical model with which to calculate how each candidate design would perform in terms of effort required to push the cart. From this information, the algorithm converges upon the familiar wheel-and-axle design, as shown in figure 2. The algorithm is iterative in that it makes a series of incremental updates to the design with each iteration. This result illustrates how the wheel-and-axle could have gradually evolved through a series of successive incremental improvements.

The output of the optimization algorithm is a monolithic structure in which the axle and wheels turn in unison. This version of the wheel-and-axle is referred to as a *wheelset*, and it is believed that this design preceded multi-body wheel-and-axle systems in which the wheels turn independently of the axle (see figure 1*d*) [15]. The monolithic nature of wheelsets meant that, compared with their multi-body counterparts, they performed poorly during turns, making it difficult to change direction. Whereas a multi-body design would allow one wheel to rotate faster than the other to cover more ground during the turn, turning on a wheelset would cause one wheel to drag along the ground, producing additional friction. A four-wheeled wagon using two wheelsets would have an even harder time negotiating turns. Here again, the context of the mine plays a crucial role. Since the mine passages were not natural geographic features, they could be made straight, thereby significantly reducing the need to turn and accommodating the use of four-wheeled carts.

Another disadvantage of the wheelset is its increased susceptibility to damage and failure. Using computer simulations, we show that in a wheelset, the wheel–axle junction experiences high stresses due in part to the torsional loads transmitted from the wheel to the axle. These loads are reduced in the multi-body wheel–axle system, which can make it more robust to damage. This factor combined with its improved manoeuverability likely led to the invention of the multi-body wheel-and-axle some 500 years after the initial invention of the wheelset [14]. This development marks the third innovation in the sequence that defines the early evolution of the wheel.

## 3. Historical and archaeological evidence for the Carpathian-roller hypothesis

**Figure 3.**
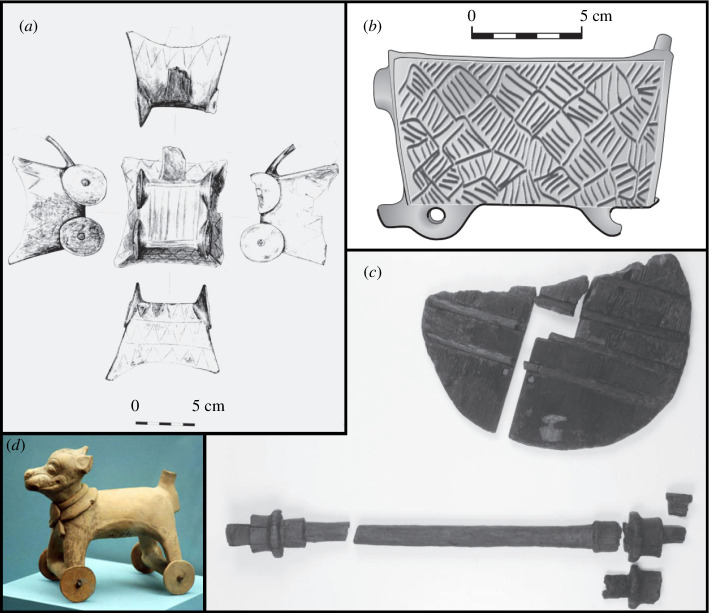
Artefacts depicting ancient wheel designs. (*a*) Sketch of a four-wheeled clay mug from the Boleráz culture [15]. (*b*) Drawing depicting a clay model of a mine cart from the Boleráz culture. A wickerwork pattern can be seen on the side panel of the cart, suggesting basketry as the likely manufacturing technique [14]. (*c*) The Ljubljana Marshes Wheel, the oldest known transport wheel, discovered in Slovenia and estimated to be between 5100 and 5350 years old [3]. The square hole in the middle of the wheel indicates that this was a wheelset in which the wheels were fixed to the axle. (*d*) A wheeled dog figurine from pre-Columbian Mesoamerica created in the eighth century AD [16].

Archaeologists excavating sites in the Carpathian Mountain region of eastern Europe have unearthed more than 150 clay models of four-wheeled wagons, all of them, judging from a big loop handle at one end, designed for use as drinking mugs (see figure 3*a*). Carbon-14 analysis dates the formation of the Boleráz culture that produced the mugs to no later than 3600 BC [15]. This makes the mugs the world’s earliest known representations of wheeled transport. But it leaves a key question unanswered: why did the Boleráz people enjoy drinking out of square mugs on wheels?

Ceremonial, even iconic, the mugs must reflect an important aspect of Boleráz culture, though one that had no apparent precursor or successor, at least in the area of tableware. Several particulars point to the inspiration for the mugs being small, wheeled baskets used to carry ore in the trenches or tunnels of copper mines. First, the mugs have wheelsets rather than wheels that revolve independently at either end of a non-revolving axle. This is consistent with a mine environment which, unlike a farmer’s field, facilitates digging and smoothing pathways that are straight and level. Second, pre-modern miners ordinarily pushed minecarts along pathways that were too constricted for draft animals, and the mugs do not show traces of yokes or other harnessing. Third, some mugs feature grooved side panels (see figure 3*b*) suggestive of the basketry shown in depictions of ancient mining [14].

The Copper Age began in the Balkan Mountains, south of the Carpathians, before the formation of the Boleráz culture. The Boleráz people had less of the metal than their southern precursors, indicating perhaps, poorer ores [14]. However, if poorer ores meant that a greater weight had to be transported from the mine to the smelter, then the invention of a wheeled basket could have been a technological breakthrough well worth celebrating with a round of drinks, at least until the wheel idea found copycats outside the mine. Notably, the earliest known transport wheels come from slightly later sites bordering the Carpathians (see figure 3*c*) where larger, more steerable carts equipped with a single wheelset were pulled by draft animals [14].

**Figure 4.**
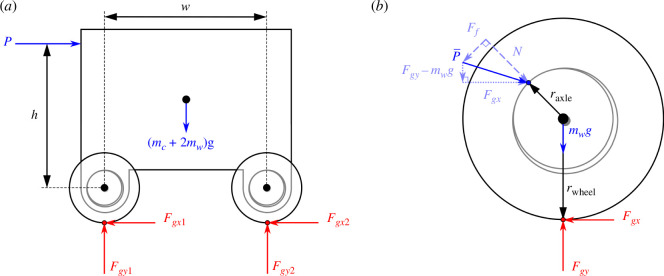
Free body diagrams for a cart with wheelsets. (*a*) A cart with a pair of wheelsets. (*b*) A single wheelset in a sliding friction bearing.

While there is no direct evidence for the use of rollers in the Carpathian region, the historical record contains multiple reports of roller-based transport being used by various civilizations that span vast distances of space and time from pre-colonial Fiji [17] to Assyria [8]. Furthermore, experimental archaeologists have successfully replicated the process of transporting megaliths using rollers in order to test their viability as an ancient method of transport [18–21]. Included among these studies is the 2001 investigation by Osenton, who showed that rollers could be fabricated using axe-based technology, and the resulting contraption enabled small groups of skilled workers to transport massive objects [21] (figure 4).

## 4. Computational analysis and design methodology

Here, we present the methods used for the analysis and generation of the optimized wheelset design. We developed several novel topology optimization methods for this purpose, the first of which is an original formulation for design-dependent variable loads in three dimensions. A variable void region, i.e. a region of empty space that is movable by the optimization algorithm, prevents the load from being applied inside the three-dimensional structure while also allowing the optimizer to place it at any location in the design domain. This enabled us to solve the wheelset design problem, which was not previously possible using any currently available topology optimization methods. Another novelty of the work is our method for achieving an axisymmetric geometry on a fixed, rectangular grid of finite elements. We use a ‘turning’ filter method which replaces the density filter matrix of standard topology optimization. Additionally, we model the orthotropic wood material in the same cylindrical coordinate system as the axisymmetric geometry filter.

### 4.1. Physics of a wheelset

Before we can perform topology optimization, we need an objective function to minimize. For this, the expression for the force required to achieve the rolling of a wheelset in a plain sliding contact bearing is derived using statics. A free-body diagram of a cart with two wheelsets is shown in figure 4*a*, where P is the applied pushing force, $m_{c}$ is the mass of the cart excluding the wheelsets, $m_{w}$ is the mass of one wheelset, g is the acceleration due to gravity and $F_{gx}$ and $F_{gy}$ are the reaction forces with the ground on each wheelset. Summing the horizontal forces gives


(4.1) $$\begin{array}{ccc} & P=F_{gx1}+F_{gx2}. & \end{array}$$

Summing the moments about the front wheelset’s contact point with the ground leads to


(4.2) $$\begin{array}{ccc} & F_{gy1}=\frac{1}{2}\left(m_{c}+2m_{w}\right)g-\frac{Ph}{w}, & \end{array}$$

and summing moments about the rear wheelset’s contact point yields


(4.3) $$\begin{array}{ccc} & F_{gy2}=\frac{1}{2}\left(m_{c}+2m_{w}\right)g+\frac{Ph}{w}. & \end{array}$$

Now, we consider one wheelset in its plain bearing by itself. The diameter of the bearing is slightly larger than the axle so that they only come into contact at a single point. Initially, with no external load applied, the bearing’s surface rests on the top of the axle. Then, as the pushing force is applied slowly, the contact point moves to the left along the axle’s surface until the friction between the bearing and axle is overcome and rolling begins [22]. In the analysis that follows, we assume the wheelsets have constant angular velocity, and therefore, they are in rotational and translational equilibrium such that all external forces and moments sum to zero.

The free body diagram for a wheelset at equilibrium is shown in figure 4*b*, where the overall force applied to the axle, $\bar{P}$ , consists of an unknown horizontal and vertical component written in terms of the mass of the wheelset, $m_{w}$ , and the reaction forces with the ground, $F_{gx}$ and $F_{gy}$ . $\bar{P}$ can also be decomposed into the components normal and tangent to the axle surface. The tangent component is the force of friction, $F_{f}$ , which depends on the normal component N as $F_{f}=\mu N$ , where μ is the coefficient of friction. We sum the moments about the centre of the axle, giving


(4.4) $$F_{gx}=F_{f}\frac{r_{axle}}{r_{wheel}}.$$

Using Pythagoras’ theorem to find an expression for N , we write the force of friction as


(4.5) $$\begin{array}{ccc} & F_{f}=\mu \sqrt{\frac{F_{gx}^{2}+{\left(F_{gy}-m_{w}g\right)}^{2}}{\mu^{2}+1}} & \end{array}$$

and substitute it into equation (4.4). Solving for $F_{gx}$ leads to


(4.6) $$F_{gx}=\frac{\left(F_{gy}-m_{w}g\right)\mu \frac{r_{axle}}{r_{wheel}}}{\sqrt{1+\mu^{2}\left(1-{\left(\frac{r_{axle}}{r_{wheel}}\right)}^{2}\right)}}.$$

Now, with an expression for the horizontal reaction force, we insert this identity into equation (4.1), eliminating $F_{gx}$ and resulting in


(4.7) $$P=\frac{\left(F_{gy1}+F_{gy2}-2m_{w}g\right)\mu \frac{r_{axle}}{r_{wheel}}}{\sqrt{1+\mu^{2}\left(1-{\left(\frac{r_{axle}}{r_{wheel}}\right)}^{2}\right)}}.$$

Substituting equations (4.2) and (4.3) into equation (4.7) for the vertical reaction forces leads to our final expression for the force required to push the cart,


(4.8) $$P=\frac{m_{c}g\mu \frac{r_{axle}}{r_{wheel}}}{\sqrt{1+\mu^{2}\left(1-{\left(\frac{r_{axle}}{r_{wheel}}\right)}^{2}\right)}}.$$

**Figure 5.**
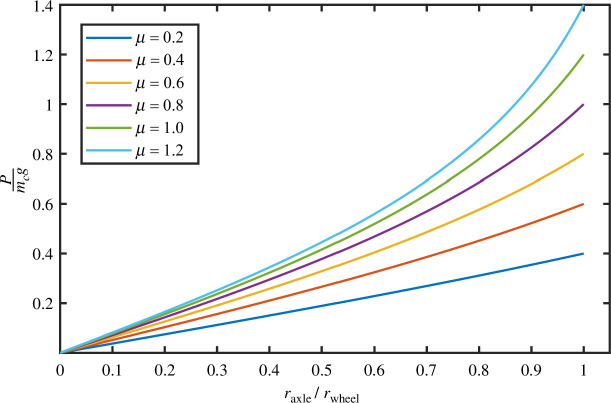
A plot showing the non-dimensionalized pushing force as a function of the axle-to-wheel diameter ratio, for several values of the coefficient of friction, μ .

The equation is plotted in figure 5. Note that this equation holds for both the wheelset and the multi-body wheel-and-axle, provided the sliding surface contains a plain bearing, as was the case for early wheels.

### 4.2. Topology optimization with a variable load

We use a three-dimensional density-based topology optimization method with a variable load location based on our previous work [23]. The variable load location is used in this problem to model the contact force between the wheelset and the cart that rests upon it. In contrast, other works modelling contact between components of a structural assembly use techniques such as spring, bar or beam element connections between coupled design domains [24–26].

A rectangular design domain of dimensions $L_{x}\times L_{y}\times L_{z}$ is discretized into a regular grid of voxels, which each contain a continuously variable density of material that can range from nearly empty space to completely solid. These densities are controlled indirectly through a vector of density design variables, 𝝆 , while the location of the applied force representing the weight of the cart is controlled directly by three variables $x_{f}$ , $y_{f}$ and $z_{f}$ representing its coordinates in space. All quantities used for the topology optimization are formulated to be continuously differentiable with respect to the design variables in order to allow for gradient-based optimization using the method of moving asymptotes (MMA) [27].

The finite element method is used to evaluate the structure defined by the design variables, treating each voxel as an eight-node hexahedral finite element. Using linear elastic physics, the finite element problem consists of solving the following linear system for the vector of nodal displacements, 𝑼 :


(4.9) $$\begin{array}{ccc} & \text{𝑲}\text{𝑼}=\text{𝑭}, & \end{array}$$

where 𝑲 is the global stiffness matrix and 𝑭 is the vector of nodal forces. We use the standard solid isotropic material with penalization (SIMP) method, which interpolates the element’s stiffness between solid and void while penalizing the stiffness-to-weight ratio of intermediate densities. The assembly of the global stiffness matrix is then given by


(4.10) $$K=\overset{N_{e}}{\underset{e}{⋀}}k_{e}^{0}(\rho_{min}+{\bar{\rho }}_{e}^{p}(1-\rho_{min}),$$

where $N_{e}$ is the number of elements, ${𝒌}_{e}^{0}$ is the element stiffness matrix of a fully solid element, ${\bar{\rho }}_{e}$ is the physical density of the element (determined from 𝝆 through filtering, described in §4.3), p is the penalization factor, and $\rho_{min}$ is the minimum allowable value for the densities (here chosen to be $1\times 10^{-6}$ ) to avoid singular global stiffness matrices. The element stiffness matrix is given by


(4.11) $$k=\int_{v_{e}}B_{e}^{T}C_{e}^{0}B_{e}dV_{e},$$

where $V_{e}$ is the volume of the element, ${𝑩}_{e}$ is the strain–displacement matrix and ${𝑪}_{e}^{0}$ is the constitutive matrix for the solid material.

**Figure 6.**
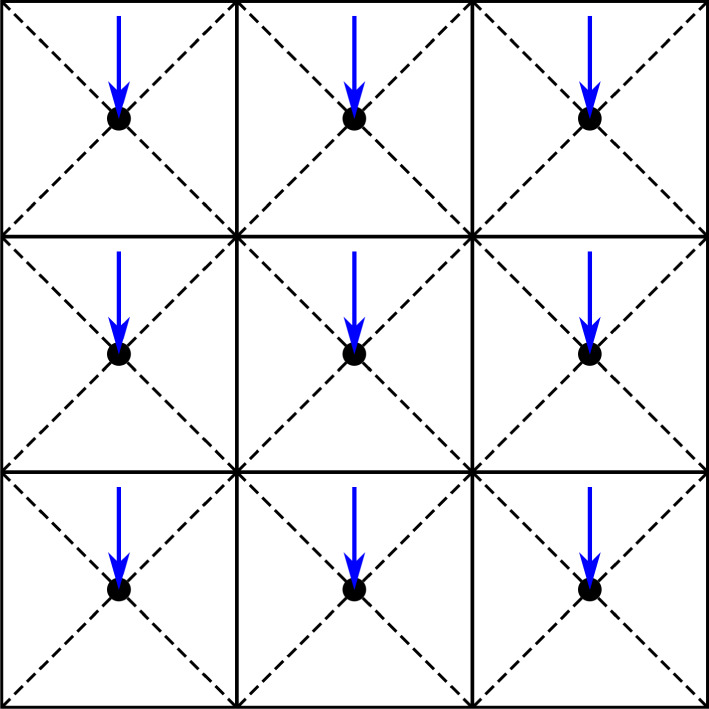
A finite element mesh with forces applied to the element centroids, which are evenly distributed to the nodes. The magnitudes (and orientations [23]) of the element forces can be varied to smoothly control the effective location of an applied load in a differentiable way.

A continuously differentiable variable load is achieved by applying forces to every element centroid, as illustrated in figure 6, and controlling the magnitudes of those forces through the design variables $x_{f}$ , $y_{f}$ and $z_{f}$ to change the effective location of a single applied load. The shape of the applied load is defined using a super-Gaussian projection function [23], which takes a distance function as input, and outputs a geometric shape of a given radius. In this case, we use the distance function for a single point to model a concentrated load, where the distance from each element centroid to the load coordinate is given by


(4.12) $$\begin{array}{ccc} & d_{e}=\sqrt{(x_{f}-x_{e}{)}^{2}+(y_{f}-y_{e}{)}^{2}+(z_{f}-z_{e}{)}^{2}}. & \end{array}$$

The distance function is passed to the super-Gaussian function, which projects it into a spherical shape of radius r defining the distribution of force magnitudes,


(4.13) $$\begin{array}{ccc} & f_{e}=A_{f}b^{-{\left(\frac{d_{e}^{2}}{r^{2}}\right)}^{S}}. & \end{array}$$

Here, $f_{e}$ is the force applied to the element centroid, $A_{f}$ is the maximum magnitude of the distributed force and S controls the sharpness of the transition of the force magnitude from $A_{f}$ to 0 . The radius parameter r determines the length from the load coordinate to a point in the transition region with magnitude $A_{f}$ divided by the base parameter b . The maximum distribution magnitude, $A_{f}$ , is chosen to give a total load equivalent to a specified number,


(4.14) $$\begin{array}{ccc} & A_{f}=\frac{P_{total}}{\sum_{e=1}^{N_{e}}b^{-{\left(\frac{d_{e}^{2}}{r^{2}}\right)}^{S}}}. & \end{array}$$

This is assumed to be a constant and is calculated only once before the optimization begins to make it easy for the user to directly specify how much load is applied.

With the spatial distribution of element centroid forces defined, the global vector of nodal forces in the finite element problem is assembled as follows:


(4.15) $$\begin{array}{ccc} & 𝑭=\overset{N_{e}}{\underset{e}{⋀}}\frac{f_{e}}{n_{n}^{e}}𝚯, & \end{array}$$

where the force at the centroid is evenly distributed to the nodes by dividing it by the number of nodes in the element, $n_{n}^{e}$ . The vector 𝚯 assigns a direction to the nodal forces by applying them to the appropriate degrees of freedom, which in this case is straight downwards,


(4.16) $$\begin{array}{ccc} & \begin{array}{ccc} & 𝚯={\left[\begin{array}{cccc} 0, & 0, & -1 & \cdots \end{array}\right]}^{T}. & \end{array} & \end{array}$$

### 4.3. Axisymmetric geometry

To limit the design space to only axisymmetric structures, a two-stage density filter is used to constrain the possible geometries to only those that can be made using a turning manufacturing process, such as a lathe. Vatanabe *et al*. have previously implemented a turning manufacturing constraint using a mapping approach [28]; however, our turning filter is an extension and modification of the casting and milling restriction filter proposed by Guest & Zhu [29]. Filtering-based manufacturing constraints have also been used for overhang limits in additive manufacturing [30,31]. In the first stage of our method, the base density design variables are mapped to an axisymmetric configuration using an $N_{e}\times N_{e}$ filter matrix 𝑾 ,


(4.17) $$\begin{array}{ccc} & \tilde{𝝆}=𝑾𝝆, & \end{array}$$

where the entries of 𝑾 are calculated as


(4.18) $$\begin{array}{ccc} & W_{ei}=\frac{w_{ei}}{\max_{e}(\sum_{i=1}^{N_{e}}w_{ei})}. & \end{array}$$

The weight factor $w_{ei}$ is non-zero only when element e is closer to the axis of symmetry than element i , and its magnitude is inversely proportional to the area of the circle centred on the axis of symmetry and intersecting the centroid of element e ,


(4.19) $$w_{ei}=\left\{ \begin{array}{ll} 1/r_{e}^{2} & \text{if }r_{e}\leq r_{i},\\ 0 & \text{if }r_{e}>r_{i}. \end{array}\right.$$

**Figure 7.**
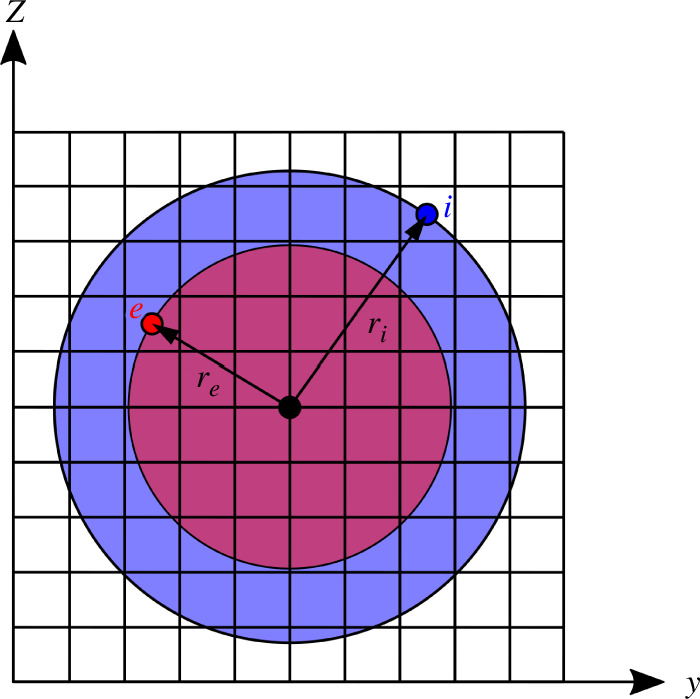
Projection domain for the turning filter for one yz layer of the three-dimensional mesh. Filtered element e is inside the unfiltered element i ’s region of influence (blue), and therefore, the filter matrix is assigned a weight at entry (e,i) that is inversely proportional to the area of the circle intersecting element e (red).

These circular regions are illustrated graphically in figure 7. The effect of applying the filter matrix is to project each unfiltered density $\rho_{i}$ to every element within the circular projection domain of that element. Each unfiltered density adds to the densities of the filtered variables, so the weight is increased closer to the axis of symmetry to compensate for fewer unfiltered variables contributing to the density of the filtered ones.

The second stage of the turning filter consists of a smoothed Heaviside step function.


(4.20) $$\overset{\approx }{\rho }=\frac{\tanh(\beta \tilde{\rho })}{\tanh(\beta )},$$

where the parameter β affects the sharpness of the step function.

**Figure 8.**
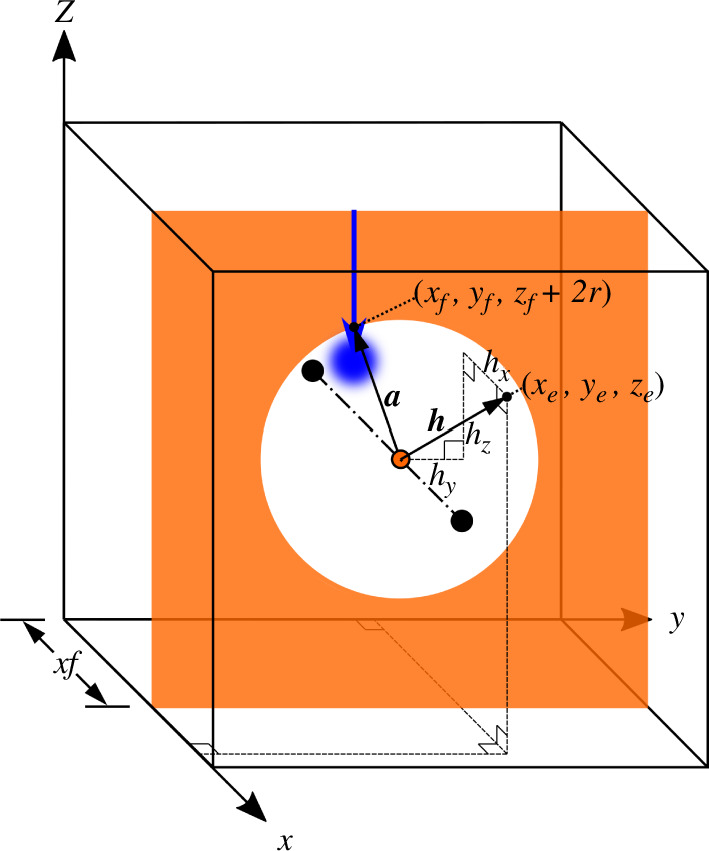
Geometry of a plane with a circular hole centred on the axis of symmetry. Used to construct the distance function for the variable void region. The location of the plane along the *x* direction and the radius of the circle depend on the coordinates of the variable load.


To avoid having the variable load penetrate the solid structure, we create an axisymmetric region of empty space that follows the location of the load. The void region begins just beyond the radius of the load application zone and extends radially outward, such that the edge of the loading zone is always applied at the solid-void interface. We use the super-Gaussian projection function to create this void space, using the minimum distance function for a yz plane with a circular hole centred on the axis of symmetry, as shown in figure 8. Defining a vector 𝒂 , which points from the centre of the void region to the point that is a distance 2r above the load coordinate,

(4.21) $$\begin{array}{ccc} & \begin{array}{ccc} & 𝒂=\left[\begin{array}{ccc} 0, & y_{f}-\frac{L_{y}}{2}, & z_{f}-\frac{L_{z}}{2}+2r \end{array}\right], & \end{array} & \end{array}$$

and a vector $𝒉=\left[\begin{array}{ccc} h_{x}, & h_{y}, & h_{z} \end{array}\right]$ , which points from the centre of the void region to an element e ’s centroid,


(4.22) $$\begin{array}{ccc} & \begin{array}{ccc} & 𝒉=\left[\begin{array}{ccc} x_{e}-x_{f}, & y_{e}-\frac{L_{y}}{2}, & z_{e}-\frac{L_{z}}{2} \end{array}\right], & \end{array} & \end{array}$$

the minimum distance from the centre of the void region geometry to that element’s centroid is given by


(4.23) $$\begin{array}{r} d_{e}=\left\{ \begin{array}{ll} |h_{x}| & \text{if }\sqrt{h_{y}^{2}+h_{z}^{2}}\geq \|\text{a}\|,\\ \sqrt{{\left(\|\text{a}\|-\sqrt{h_{y}^{2}+h_{z}^{2}}\right)}^{2}+h_{x}^{2}} & \text{if }\sqrt{h_{y}^{2}+h_{z}^{2}}<\|\text{a}\|. \end{array}\right. \end{array}$$

The distance function is then projected to a density field of the same radius as the load application region before being subtracted from a uniform field of 100% density,


(4.24) $${\hat{\rho }}_{e}=1-(1-\rho_{min})b^{-{\left(\frac{d_{e}^{2}}{r^{2}}\right)}^{S}},$$

where the coefficient $\left(1-\rho_{min}\right)$ is used to maintain the minimum density value. Finally, using a smooth minimum function, the void region is combined with the filtered densities to give the physical density field that represents the actual design on which the finite element simulations are based,


(4.25) $${\bar{\rho }}_{e}={\left(\frac{{\overset{\approx }{\rho }}_{e}^{Q}+{\hat{\rho }}_{e}^{Q}}{2}\right)}^{\frac{1}{Q}}.$$

Here, Q is a negative number such that the smooth minimum function approaches the true minimum as the magnitude of Q increases.

### 4.4. Orthotropic material properties

We use orthotropic material properties in a cylindrical coordinate system about the axis of symmetry. Topology optimization using orthotropic materials is not new and has been used for applications such as continuous fibre composites [32]. In our application of a structure made from a single piece of wood, the material coordinates only need to be defined once before the optimization begins. Wood’s three mutually perpendicular axes are the longitudinal, radial and tangential directions. The longitudinal direction runs parallel to the axis of symmetry, the radial direction is normal to the log’s growth rings, and the tangential direction is parallel to the rings [33]. We use the approximate material properties of spruce trees obtained from data in [33] and shown in table 1. The remaining Poisson’s ratios $\nu_{23}$ , $\nu_{31}$ and $\nu_{21}$ are calculated as

**Table 1.** Material properties.

$E_{1}$	10 GPa
$E_{2}$	0.5 GPa
$E_{3}$	1.0 GPa
$G_{12}$	0.9 GPa
$G_{31}$	0.9 GPa
$G_{23}$	0.07 GPa
$\nu_{12}$	0.46
$\nu_{13}$	0.40
$\nu_{32}$	0.48
density	400 kg m^−^ 3


(4.26) $$\begin{array}{ccc} & \nu_{ij}=\nu_{ji}\frac{E_{i}}{E_{j}}. & \end{array}$$

The constitutive matrix for a solid element in a local coordinate system is


(4.27) $$\begin{array}{ccc} & \begin{array}{ccc} & {𝑪}^{0}={\left[\begin{array}{cccccc} 1/E_{1} & -\nu_{21}/E_{2} & -\nu_{31}/E_{3} & 0 & 0 & 0\\ -\nu_{12}/E_{1} & 1/E_{2} & -\nu_{32}/E_{3} & 0 & 0 & 0\\ -\nu_{13}/E_{1} & -\nu_{23}/E_{2} & 1/E_{3} & 0 & 0 & 0\\ 0 & 0 & 0 & 1/G_{12} & 0 & 0\\ 0 & 0 & 0 & 0 & 1/G_{23} & 0\\ 0 & 0 & 0 & 0 & 0 & 1/G_{31} \end{array}\right]}^{-1}, & \end{array} & \end{array}$$

where the subscript 1 is the longitudinal direction, 2 is tangential and 3 is radial. Now, for each element in the mesh, the constitutive matrix is rotated an angle θ so that the radial direction points away from the axis of symmetry,


(4.28) $$\begin{array}{ccc} & {𝑪}_{e}^{0}(\theta )=𝑹(\theta ){𝑪}^{0}{𝑹}^{T}(\theta ). & \end{array}$$

**Figure 9.**
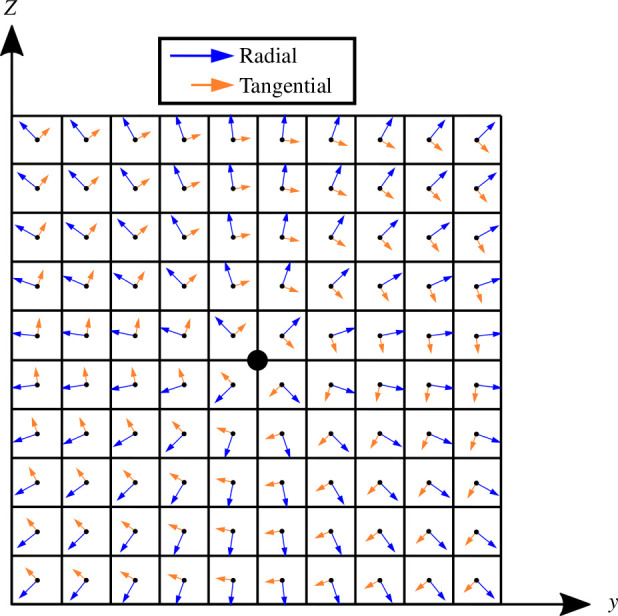
Local coordinates of each element in one layer of the three-dimensional finite element mesh. Each element’s orthotropic material properties are oriented in a cylindrical coordinate system about the axis of symmetry.

The rotation matrix 𝑹(θ) is


(4.29) $$\begin{array}{ccc} & \begin{array}{ccc} & 𝑹(\theta )=\left[\begin{array}{cccccc} 1 & 0 & 0 & 0 & 0 & 0\\ 0 & c^{2} & s^{2} & 2cs & 0 & 0\\ 0 & s^{2} & c^{2} & -2cs & 0 & 0\\ 0 & -cs & cs & c^{2}-s^{2} & 0 & 0\\ 0 & 0 & 0 & 0 & c & -s\\ 0 & 0 & 0 & 0 & s & c \end{array}\right], & \end{array} & \end{array}$$

where c=cos⁡θ and s=sin⁡θ . The rotated material property orientations in a yz layer of the mesh are shown in figure 9.

### 4.5. Numerical implementation

The optimization task is formulated as a multi-objective problem where we seek to minimize the force required to move the cart as well as the structural compliance, given a fixed amount of material to work with. The pushing force is given by equation (4.8), and the compliance, C , is the dot product of the load and displacement vectors,


(4.30) $$\begin{array}{ccc} & C={𝑭}^{T}𝑼. & \end{array}$$

The amount of material is constrained using the volume fraction,


(4.31) $$\begin{array}{ccc} & V_{f}=\frac{\sum_{e=1}^{N_{e}}{\bar{\rho }}_{e}V_{e}}{L_{x}L_{y}L_{z}}. & \end{array}$$

Our optimization problem is then written as aC


(4.32) $$\begin{array}{rlrlrl} & \underset{\rho ,x_{f},y_{f},z_{f}}{\text{minimize}} & & P(y_{f},z_{f})+aC(\rho ,x_{f},y_{f},z_{f})\\ & \text{subject to:} & & V_{f}(\rho ,x_{f},y_{f},z_{f})\leq V_{f}^{*},\\ & & & \rho_{min}\leq \rho_{e}\leq 1, & & e=1,\ldots ,N_{e},\\ & & & r\leq x_{f}\leq L_{x}-r,\\ & & & r\leq y_{f}\leq L_{y}-r,\\ & & & \frac{L_{z}}{2}\leq z_{f}\leq L_{z}-r, \end{array}$$

where P is the pushing force evaluated using equation (4.8) with the axle radius calculated as the distance from the point of load application to the axis of symmetry, $r_{axle}=\sqrt{(y_{f}-L_{y}/2{)}^{2}+(z_{f}-L_{z}/2{)}^{2}}$ , and the coefficient $a=1\;{\text{m}}^{-1}$ , which ensures dimensional consistency across terms within the objective function. Bounds on the design variables are set such that the densities can vary between nearly void and solid, and the applied load can move anywhere in the upper half of the domain.

**Figure 10.**
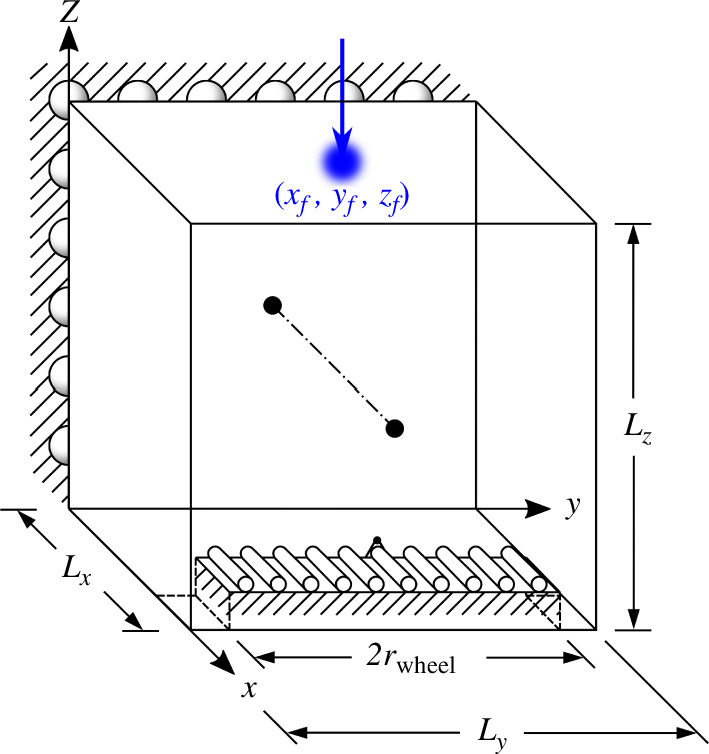
The wheelset optimization problem’s domain and boundary condition set-up. Only half of the domain is simulated by taking advantage of the plane of symmetry at the wheelset’s centre. The ground the wheel rests on is modelled by a rectangular region of boundary conditions within the domain. The variable load is initialized in an arbitrary position away from where it is expected to move to in the optimal design.

Taking advantage of the symmetry of the problem, only one-half of the wheelset is modelled and optimized. The symmetry is modelled using roller boundary conditions by fixing the displacements in the x direction on the face of the domain at x=0 . Boundary conditions representing the ground that the wheel rests on are placed at 10% of the domain’s width above the lower face and away from the side faces so that the wheel can be supported by more than a single point by sinking into the ground slightly. The ground extends 20% the width of the domain from the face at $x=L_{x}$ , and the x and z displacements are fixed on this area. A single node at the middle of the inner edge is also fixed in the y direction to fully constrain the problem by preventing rigid body translation. The variable load is initialized at the top face and near the x=0 face, with a magnitude equivalent to one-quarter of the weight of a 100 kg cart. The domain and boundary condition set-up described here is illustrated in figure 10.

The program is implemented in C++ using the PETSc library [34,35], based on the code originally written by Aage *et al*. [36]. Using a mesh of 104×104×104 (1,124,864) eight-node hexahedral (cube) elements and the parameters shown in table 2, we ran the problem on the Illinois Campus Cluster using 32 nodes and 640 processes. The optimization search was considered to have converged when the maximum change in the density design variables was less than 0.001. The optimization completed in 9 h and 38 min after 776 iterations, with the final design shown in figure 2.

**Table 2.** Optimization parameters.

$L_{x}$	37.5 cm
$L_{y}$	37.5 cm
$L_{z}$	37.5 cm
$P_{total}$	245 N
$V_{f}^{*}$	3%
p	3
β	8000
b	2
r	1.875 cm
S	4
Q	−8
$\rho_{min}$	$1\times 10^{-6}$
Move Limits: ρ	0.05
Move Limits: $x_{f},y_{f},z_{f}$	0.9375 cm

### 4.6. Failure analysis

Failure of orthotropic materials, like wood, can be predicted by performing a stress analysis and computing a failure index. Many different failure criteria for orthotropic materials exist, such as the Tsai–Hill [37], Hoffman [38] or Tsai–Wu [39] failure criteria. The Tsai–Hill index has been found to work adequately well for predicting wood failure [40,41]. We use the approximate strength properties of spruce wood [33], shown in table 1, to analyse the wheelset design. The Tsai–Hill failure index is given by


(4.33) $$\begin{array}{ccc} & {\left(\frac{\sigma_{1}}{\sigma_{1}^{f}}\right)}^{2}-\frac{\sigma_{1}\sigma_{2}}{{\left(\sigma_{1}^{f}\right)}^{2}}+{\left(\frac{\sigma_{2}}{\sigma_{2}^{f}}\right)}^{2}+{\left(\frac{\tau_{12}}{\tau_{12}^{f}}\right)}^{2}<1, & \end{array}$$

where the superscript *f* denotes a failure stress material property. Violation of the inequality indicates that failure has occured.

Linear elastic finite element analysis was performed in Ansys using the orthotropic material properties from tables 1 and 3 in a cylindrical coordinate system about the axis of symmetry. Two models were created: one as a single solid component, and a second where the axle is separated from the wheel to create a wheel-and-axle assembly. Only half of the wheelset was modelled to save on computational cost, and a portion of the bottom of wheel was cut off to apply a fixed boundary condition simulating a contact patch. A vertical downward load of 2000 N was applied to the face of the axle next to the wheel where the load application zone was applied in the topology-optimized design. Using equation (4.8) with the dimensions of the model ( $r_{axle}=0.037$ m and $r_{wheel}=0.17$ m) gives a corresponding pushing force of 986.5 N, which was applied as a horizontal load on the same face of the axle. In the wheel-and-axle assembly model, a frictionless contact condition was applied to the bearing surface. Each model was meshed with approximately 60,000 quadratic tetrahedral elements, with increased refinement in the axle.

**Table 3.** Strength properties.

$\sigma_{1}^{f}$	35.7 MPa
$\sigma_{2}^{f}$	3 MPa
$\sigma_{3}^{f}$	3 MPa
$\tau_{12}^{f}$	6.7 MPa
$\tau_{23}^{f}$	6.7 MPa
$\tau_{13}^{f}$	6.7 MPa

## 5. Results

**Figure 11.**
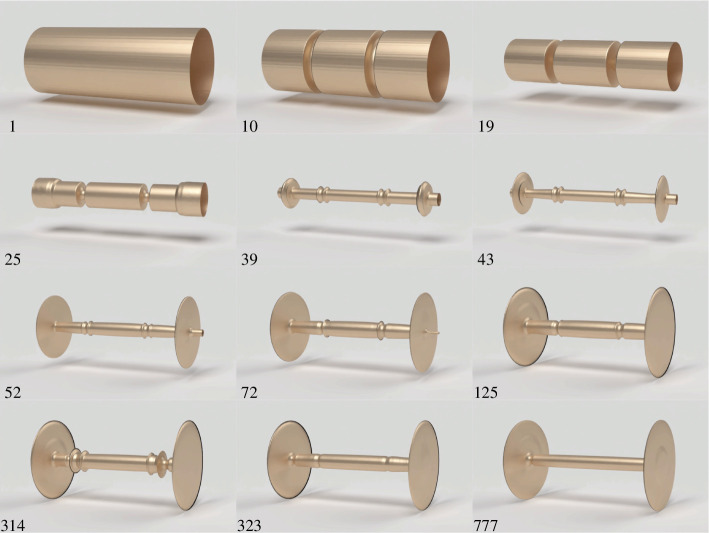
Progression of the wheelset design during execution of the topology optimization algorithm. The iteration numbers are given in the bottom left corner of each image.

Figure 11 shows the progression of the wheelset structure during the execution of the topology optimization algorithm. The design search begins with a solid prismatic roller. From this starting point, two grooves appear and grow progressively deeper. This is followed be the narrowing of the central portion of the roller to form an axle, and the expansion of the outer discs to form wheels. The resulting wheelset design maximizes mechanical advantage and structural stiffness.

**Figure 12.**
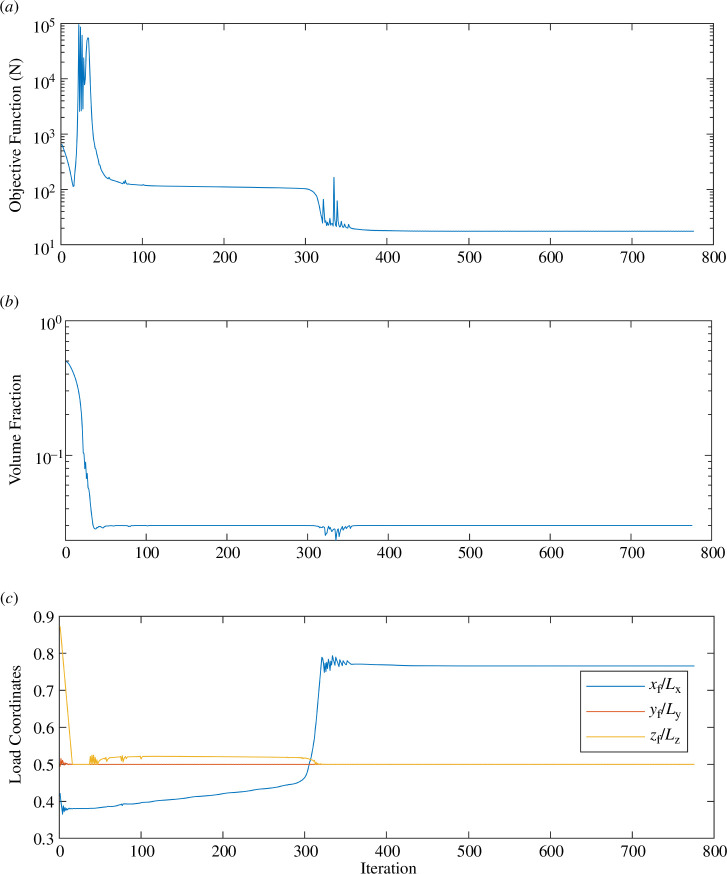
Convergence histories for the optimization of the wheelset shown in figure 11.

Figure 12 shows the optimization convergence history for the result shown in figure 11. The first plot shows the convergence of the combined objective function, which is defined as the sum of the pushing force plus the structural compliance. The second plot contains the convergence history of the spatial coordinates of the point of load application. There is a noticeable dip in the objective function beginning around iteration 300, when the optimizer pushes the contact point outward towards the wheels, causing a reduction in structural compliance. Note also that both convergence plots contain regions where the objective and constraint function values oscillate. These regions reflect the inherent difficulty of numerically solving a mixed topology optimization problem that combines density variables with explicit variables representing the load coordinates. This combination negatively impacts the convergence of the algorithm. Here, we mitigate this effect by imposing move limits on the design variables.

The pushing force and structural stiffness objectives are in conflict. This can be shown by plotting a Pareto front [42,43] of the optimization problem described in equation (5.1). The objective function is modified by multiplying the pushing force ( P ) and the compliance ( C ) terms by a variable weighting factor, α . The compliance term is also multiplied by a scaling factor of $10^{6}$ m^−1^ so that the two terms are similar in magnitude and have consistent units. Note that each point on the resulting Pareto front represents a separate optimization in which we have minimized the objective function given in equation (5.1), while enforcing all the previous optimization constraints described in (4.32)


(5.1) $$\begin{array}{ccc} & \begin{array}{ccc} & \begin{array}{cccc} & \underset{𝝆,x_{f},y_{f},z_{f}}{\text{minimize}} & & \alpha P+(1-\alpha )C\times 10^{6}{\text{m}}^{-1}. \end{array} & \end{array} & \end{array}$$

**Figure 13.**
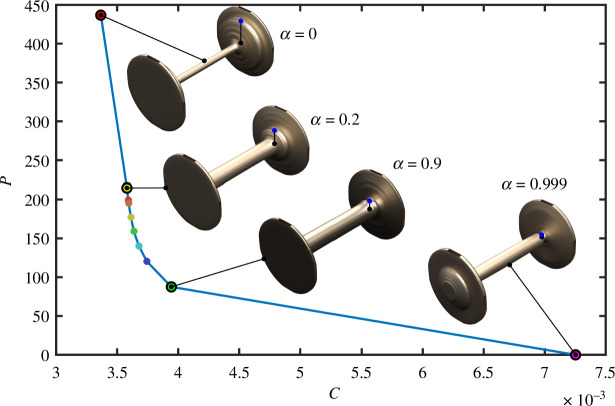
The Pareto front of the weighted-objective optimization problem (equation 5.1). The x - and y -axes are expressed in standard SI units of N and N m, respectively. In the wheelset images, the blue dots indicate the contact points (i.e. the location of load application), and the black dots indicate the location of the axis of rotation, therefore the distance between these two dots is inversely proportional to the mechanical advantage.

Using a resolution of 80×80×80 elements and a volume fraction limit of 10%, we solved the optimization for several values of α ranging from 0 to 0.999. When α=0 , the optimization is purely a compliance minimization problem. As α approaches 1, the pushing force becomes more heavily weighted. In this case, the optimizer drives the pushing force to its minimum possible value of zero. The values of the pushing force P and the structural compliance C are recorded for each solution, and the Pareto front is plotted in figure 13 along with images of some of the optimal designs. It can be seen from the Pareto plot that if one objective decreases, the other increases, hence the trade-off.

**Figure 14.**
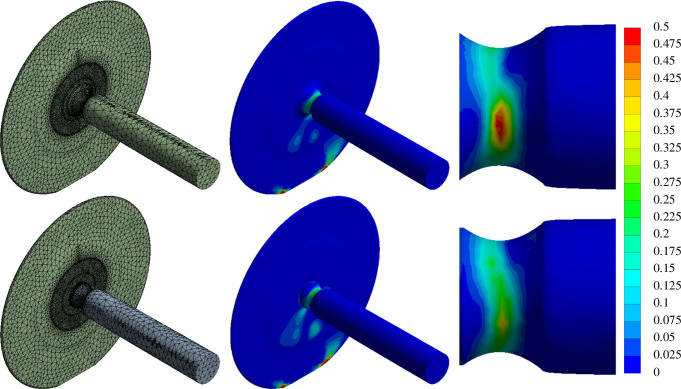
Finite element simulation of the wheelset (top row) versus the multi-body wheel-and-axle (bottom row). The contours show the Tsai–Hill stress distribution within the volume of the structure. The results show that the multi-body wheel-and-axle system experiences reduced stress and is therefore less likely to sustain material damage and structural failure.

Figure 14 shows a comparison of the stress distributions in wheelset and multi-body mechanisms with identical geometries and loading conditions. In the monolithic wheelset, the peak Tsai–Hill failure index on the axle is 0.56, while on the wheel-and-axle assembly, it only reaches 0.43 because of the frictionless bearing preventing the transfer of a torsional stress between the wheel and axle. This result supports the hypothesis that a multi-body wheel-and-axle system would have been more robust to damage than a wheelset structure. Note that the optimization results shown in figures 11–13 did not account for the Tsai–Hill failure index and that failure analysis was performed as a post-processing step to analyse the optimized designs.

## 6. Significance and implications of our findings

These results indicate that our proposed sequence for the progression of the wheel and axle offers a plausible *descent path* along which the wheel could have evolved as its users sought more energy-efficient designs. Our findings also demonstrate the critical role that environmental factors played in the creation of wheeled technology. The unique features of the mine environment accentuated the advantages of the wheelset over its predecessor while negating its most significant disadvantage: the inability to turn. Despite its shortcomings, the wheelset played a crucial role in the evolution of the wheel due to its conceptual proximity to the roller, which served as a technological bridge.

It should be noted that our investigation does not span the full breadth of the wheel’s historical development, and the wheel did not stop evolving with the advent of the multi-body wheel-and-axle. The technology came full circle in 1869 with the invention of radial ball bearings [44], which, ironically, reintroduced bilateral rolling but solved the issue of spent rollers by enclosing the rollers (i.e. bearings) within a cage. Nor do we wish to imply that this was the only time in history that the wheel was independently discovered. The indigenous peoples of the Americas also had knowledge of wheeled locomotion as evidenced by the presence of wheeled figurines from the eighth century BC in what is now southern Mexico (see figure 3*b*) [16,45]. However, the Boleráz wheels represent the earliest known instance of wheeled transport, and they are a likely source of the wheel’s early proliferation throughout the Old World.

This chapter in human history runs counter to the popular belief that technologies arise abruptly from the sudden epiphany of a lone inventor. Consequently, some scholars have embraced the other extreme, claiming that the wheel had no origin point and no inventor, but rather it developed gradually across a broad geographical area [5]. A similar theory has also been applied broadly to technology in general [46]. Our investigation modifies this theory and adds several new insights. The wheel evolved over a period spanning many centuries, but this evolution was punctuated by discrete innovations. We also find that, much like biological organisms, the wheel evolved from an earlier technology from which it inherited certain advantageous traits, and this evolution was fundamentally linked to the local environment. Lastly, our study is an example of how we can leverage the techniques of design science and computational mechanics to uncover new knowledge about episodes from our distant past for which there is no written record, and our only clues are the artefacts left behind by the designers who came before us.

## Data Availability

All data and computer code used to generate the results presented in this study are publicly available via the following permanent repository [47].
